# Checking Perspiration

**Published:** 1879-07

**Authors:** 


### CHECKING PERSPIRATION.

A MAN walked rapidly along the street on a warm day, and while perspiring stepped into a barber's shop to be shaved, and pulling off his coat waited for his turn; but the room was cool, a chill sensation came over him before the barber completed his operations; a fever followed, then inflammation of the lungs, ending, in a few days, in the death of the good and universallv loved Bishop McIlwaine.

